# Poster Session II - A251 THE EFFECT OF *LACTICASEIBACILLUS RHAMNOSUS* STRAIN C51 IN THE PREVENTION OF COLITIS IN HIGH PROTEOLYTIC IBD MICROBIOTA COLONIZED MICE

**DOI:** 10.1093/jcag/gwaf042.251

**Published:** 2026-02-13

**Authors:** B Bowerman, A Hann, X Mas Orea, M Bording-Jorgensen, S Gontier, A Aucouturier, I Naas, P Bercik, M Surette, H Galipeau, P Moayyedi, P Langella, E F Verdu

**Affiliations:** McMaster University, Hamilton, ON, Canada; McMaster University, Hamilton, ON, Canada; Medecine, McMaster University Faculty of Health Sciences, Hamilton, ON, Canada; McMaster University, Hamilton, ON, Canada; Microbiologie de l’Alimentation au Service de la Sante, Jouy-en-Josas, Île-de-France, France; Microbiologie de l’Alimentation au Service de la Sante, Jouy-en-Josas, Île-de-France, France; Microbiologie de l’Alimentation au Service de la Sante, Jouy-en-Josas, Île-de-France, France; McMaster University, Hamilton, ON, Canada; McMaster University, Hamilton, ON, Canada; McMaster University, Hamilton, ON, Canada; McMaster University, Hamilton, ON, Canada; Microbiologie de l’Alimentation au Service de la Sante, Jouy-en-Josas, Île-de-France, France; McMaster University, Hamilton, ON, Canada

## Abstract

**Background:**

Microbial proteolytic imbalance occurs in about 30% of patients with inflammatory bowel disease (IBD), yet drivers and therapies targeting this dysfunction remain unknown. A high salt diet (HSD) is associated with increased colitis risk and higher predicted microbial proteases. Whether HSD-induced high proteolytic activity (PA) increases colitis risk or responds to anti-proteolytic therapy remains unknown.

**Aims:**

1. To determine whether HSD-induced high PA (HPA) microbiota worsens colitis severity.

2. To test whether elafin-producing *Lacticaseibacillus rhamnosus* (LrC51) prevents spontaneous colitis in mice colonized with HPA-IBD microbiota.

**Methods:**

Adult germ-free (GF) C57BL/6 mice were colonized with low PA-IBD microbiota that induced a switch to HPA after HSD (HSD “responder”) or IBD microbiota that did not respond to HSD (“non-responder”). Mice were fed either a control diet (CD) or an isocaloric HSD supplemented with 4% NaCl (HSD). After three weeks, all mice were fed a CD and subjected to 1.5% dextran sodium sulfate (DSS) for 5 days followed by 2 days of water. Fecal blood was evaluated daily during colitis.

Adult GF *Il10*^-/-^ mice were colonized with HPA-IBD microbiota and fed a CD. After 3 weeks, mice received daily oral gavages with LrC51, wild-type (WT) control (Lcr35), or PBS+15% glycerol for 2 weeks. Cecal PA was measured (azocasein & FITC-elastin assays), and terminal ileum and colon was collected for histological analysis.

**Results:**

In mice colonized with responder IBD microbiota, a HSD led to more severe fecal blood on average versus both HSD-responder and non-responder colonized mice fed a CD (p = 0.08 and p = 0.02, respectively). In *Il10*^-/-^ mice colonized with HPA-IBD microbiota, LrC51 reduced microscopic scores in the colon and ileum compared with both WT (p < 0.001) and vehicle control (p < 0.01). LrC51 treated mice had lower overall proteolytic activity in the cecum (p = 0.04) compared to WT controls.

**Conclusions:**

Mice colonized with HSD-responder microbiota and fed a HSD prior to low-grade DSS develop severe clinical signs of colitis. In the spontaneous *Il10*^-/-^ model, the anti-protease producing probiotic LrC51, ameliorated colitis in HPA-IBD microbiota colonized mice. This probiotic is biocontained and its WT form is commercially available, which could rapidly translate to application in humans with IBD.

**Funding Agencies:**

CIHR

